# Patient characteristics and dispatch responses of urinary tract infections in a prehospital setting in Copenhagen, Denmark: a retrospective cohort study

**DOI:** 10.1186/s12875-022-01915-4

**Published:** 2022-12-10

**Authors:** Jeske Verhoeven, Helle Collatz Christensen, Stig Nikolaj Blomberg, Simone Böbel, Mirjam Scholz, Thomas Krafft

**Affiliations:** 1 Faculty of Health, Medicine and Life Sciences (FHML), Maastricht University, Universiteitssingel 60, 6229 ER Maastricht, the Netherlands; 2 Emergency Medical Services Copenhagen, University of Copenhagen, Telegrafvej 5, 2750 Ballerup, Denmark

**Keywords:** Urinary tract infection, Cystitis, Emergency medical services, Emergency medical dispatch, Dispatch response, Emergency response, Out-of-hours primary care

## Abstract

**Background:**

Urinary tract infection (UTI) is particularly common in young women and the elderly. The Emergency Medical Services (EMS) in Copenhagen, Denmark can be reached by calling either of two dedicated telephone lines: 1–1-2 in case of an emergency and 1813 during general practitioner’s (GP) out-of-office hours (OOH). This study investigated characteristics of patients with symptoms of UTI calling the Copenhagen EMS and the response they received.

**Methods:**

A retrospective observational cohort study was conducted in which 7.5 years of telephone data on UTI from the EMS in Copenhagen were analyzed. Descriptive statistics and multinomial logistic regression were used to analyze patient characteristics, the timing of the incident and response. Patients’ age and gender were assessed and the use of urinary catheters, the timing of the incident, and the impact on the response were evaluated.

**Results:**

A total of 278.961 calls were included (78% female, mean age 47), with an average of 120 patients with UTI symptoms calling each day. Most people contacted the 1813-medical helpline (98%) and of those, the majority were referred to the emergency department (ED)(37%). Patients were more likely to be referred to the ED during the weekend compared to a weekday and less likely during OOH compared to in-office hours (IH). Patients with a urinary catheter were more likely to receive specialized care referred to as ‘other’. For the smaller proportion of patients calling 1–1-2, most people got a B (urgent) response (1.5%). The most likely response to be given was an A (emergency) or F (non-emergency) response during OOH compared to IH and on weekends compared to weekdays. Patients with a urinary catheter were more likely to receive a D (unmonitored transport) response.

**Conclusions:**

Since 2015, there was a decrease in 1813 antibiotic prescription rates and a subsequent increase in referral to the ED of UTI patients. Patients were referred less to the ED during OOH as they were likely to be sent to their GP the next day. During the weekend, patients were referred more to the ED for the likely reason that their GP is closed.

**Supplementary Information:**

The online version contains supplementary material available at 10.1186/s12875-022-01915-4.

## Background

Urinary tract infection (UTI) is a prevalent public health phenomenon, occurring in about 11 % of the population, and is especially common in women, where about 50% of all women experience at least one infection during their lifetime [1–3]. The rate of UTI is highest in premenopausal and sexually active women. Men generally demonstrate lower rates of UTI, namely 5 to 8 cases per 10,000 young and middle-aged men and 20–50% in men older than 50 years. The latter are mostly affected by it due to underlying causes such as prostate enlargement or catheterization [2]. Moreover, UTI is among the most common infectious cause of hospitalization among the senior population [3, 4]. Among the elderly, 10% of the females over the age of 65 state that they have had symptoms of a UTI within the last year [5].

In the Danish regions outside of the greater Copenhagen Region, GP cooperatives are responsible for out-of-office (OOH) care. A study conducted on the OOH services in Central Denmark Region found that almost 60 % of the OOH telephone resulted in a telephone consultation [6]. This is in line with the findings of a study on guideline adherence in OOH services in Europe, which showed that uncomplicated UTIs in Denmark are often treated via telephone consultations. The authors also found that cystitis is the most frequent diagnosis in telephone consultations [7]. In accordance, this diagnosis is also highly prevalent on the list of referrals to face-to-face contact [6]. Furthermore, studies on antibiotic prescription found significantly higher rates of prescription in Denmark (80 prescriptions/1.000 inhabitants) than comparable countries such as Sweden, England and the Netherlands (28, 31 and 30 prescriptions, respectively) [8–12].

Previous research on OOH primary care in the Netherlands – a country with a strong resemblance to Denmark in terms of healthcare systems [8] – showed that the majority of UTI patients calling the service visited the OOH center for evaluation. However, treatment rates were similar for patients visiting the center as for patients that only had a telephone consultation. In total, almost three-quarters of the patients with UTI symptoms contacting the OOH service received antibiotics [13]. Another study on primary care services in Sweden assessed the difference in antibiotic treatment between IH and OOH. The findings show that of the prescribed antibiotics, 16% were commonly used for UTIs and that UTIs were more common during OOH, despite only 12% of all infection visits occurring during OOH. Finally, the rate of antibiotic prescription was 9% higher during OOH compared to IH [9]. Similar findings were generated in a study on prescription rates between IH and OOH primary care in the Netherlands, which discovered that antibiotic prescription differed from 62% during IH to almost 94% during OOH [11].

Given the information that uncomplicated UTIs are often treated through telephone consultations in OOH services [6], we hypothesize that the antibiotic prescription rate during this response type will be the most common treatment of people with UTI. However, considering that a fraction of the people calling the emergency number 1–1-2 and 1813-medical helpline will be people with a complicated manifestation of UTI – such as men, pregnant women, and patients with a urinary catheter [14] – there is likely to be a difference in response type for patients who show different characteristics, such as age, gender and comorbidity.

Therefore, to investigate these notions, this study aims to assess the characteristics of patients with symptoms of UTI calling the EMS in Copenhagen and the response they received from the dispatcher. Here, patient characteristics are presented by quantifying the distribution of age and gender and assess the use of urinary catheters and its impact on the response. In addition, there is a need to assess the timing of the incident and how this influences the response from the EMS, as previous studies showed that there are fluctuations in response between IH and OOH care [9, 11, 15]. Accordingly, the rationale is to differentiate between patients with and without urinary catheter to represent a form of complicated infections. Moreover, differentiation in timing of the calls made, such as differences in response to telephone calls made during the week and the weekend and calls made during IH and OOH needs to be identified.

## Methods

### Study design

A retrospective observational cohort study was conducted. Anonymized data on telephone calls made to the EMS in Copenhagen during a period of approximately 7.5 years (December 10th, 2013 – May 7th, 2021) were collected.

### Setting

EMS Copenhagen covers a population of 1.8 million people both from urban and rural areas and with different socio-economic status [16]. In 2019, more than 1 million calls were made to the 1813-medical helpline and around 130.000 calls were made to the 1–1-2 emergency number [17]. There are two medical helplines in the Copenhagen region that patients can use to contact the emergency medical services (EMS) by telephone. In case of an emergency or during out-of-office hours (OOH) of their general practitioner (GP), patients with UTI symptoms can call the emergency number 1–1-2 or OOH number 1813 [18]. The consultation of OOH primary care by patients has been increasing over the past years, which is a direct result of higher demand for non-urgent contacts alongside extensive opening hours and improvement in services [8]. Through the 1813-medical helpline, Danish citizens are in direct contact with specially trained physicians and nurses, who answer the telephone and perform triage 24/7, with the help of a decision support tool [17]. During the in-hours (IH) of GPs in Denmark, nurses and GPs to a lesser extent operate the 1813-medical helpline and during the OOH timeline – which is from 16:00 till 8:00 in Copenhagen – a higher number of GPs support them by concurrently answering and responding to calls and by giving them a second opinion where needed. For the 1–1-2 emergency medical helpline, it is nurses and paramedics answering the calls. Nurses switch between the two telephone lines to be knowledgeable in both systems. Furthermore, the systems are integrated to a certain extent, which for instance means that it is also possible for nurses or GPs at the 1813-medical helpline to switch to the emergency protocol (Danish Index) to dispatch an ambulance [17]. In case of a suspected UTI, dispatchers rely on their medical training and the Computer-Aided Dispatch (AID) system to guide them through the call and reach a response. They can give telephone advice, direct patients to a hospital-based acute care department, schedule a home visit [18], or prescribe a certain selection of antibiotics (GPs only) [19]. Diagnoses are not given over the telephone, nor are they registered in the system. The system merely registers the response given by the dispatcher.

### Data collection

Administrative data from the telephone registrations in Computer-Aided Dispatch from Logis were collected, which is an intelligent online decision support tool, helping emergency services to better serve their patients and communities, which is used by the Copenhagen EMS [16].

To identify the UTI cases in the data base, the following steps were applied. Firstly, records from the criteria text, the automatic click system used in the 1813-medical helpline, were screened on ‘UVI’ (short for urinevejsinfektion, Danish for UTI). Additionally, data from the notes made by dispatchers on the cause of the telephone call and other written input from open text fields in the database were screened on the words ‘UVI’ and ‘blærebetændelse’ (Danish for cystitis). Duplicates were removed based on caller ID.

The telephone triage data consisted of information on gender, age, which number was called (1–1-2 or 1813), response type (treatment plan, varying from telephone consultation to ED referral), cause and time of calling, and patient use of a catheter. The latter was retrieved similarly to the UTI data, namely in the criteria text of the UTI records using the words ‘kateter’ (catheter) and ‘KAD-relaterede problemer’ (catheter-related problems), and in the cause text and written input in open text fields using the words (and variants of them) ‘kateter’, ‘KAD’, ‘topkateter’ (suprapubic catheter), ‘urinposen’ (urine drainage bag), and ‘blærekateter’ (bladder catheter). After removing duplicates, records from the introduction of the 1813-medical helpline until the start of the data collection were screened and accumulated in a data frame using SAS Enterprise Guide 7.1.

### Data analysis

Both the 1–1-2 and the 1813 responses were grouped into categories as follows: For 1–1-2 response, the five general responses according to the Danish Index (Table 1) were used as the main categories, whereas the remainder was grouped as ‘other’. The 1813 response was also grouped into six categories, based on the type and prevalence of the responses. ‘Selfcare’, ‘GP next day’, ‘prescription’ and ‘hospital admission’ were response categories on their own, whereas ‘referral to ED’ is a combination of three different emergency department (ED) referral categories, which includes admission. ‘Hospital admission’ refers to a direct admission into a hospital department other than the ED, such as urology or gynecology (in case of pregnancy). The remaining categories, which includes several categories with various meanings, such as referral to a special care physician or a home visit, was grouped as ‘other’. During a call, patients can only be assigned to one category. If more categories are applicable, the most important category is chosen.

**Table 1:** Categories of the Danish index for emergency care

Category	Meaning
A	Acute – potentially life threatening
B	Urgent – not assessed as life threatening
C	Planned – not acute, but need to be monitored and treated in the ambulance
D	Supine transport – transportation while lying down, but without need to be monitored and treated
F	Non-emergency – other services as home visit, advice, etc.

Descriptive statistics were performed based on frequencies [20]. Patient characteristics were summarized in a table. In addition to the overall prevalence, the 1–1-2 and 1813 response was plotted throughout the years to understand the changes over time. Furthermore, the patients’ age was plotted with stratification for gender to visualize the distribution of age for both men and women. Additionally, the hour and the day of the call were plotted to visualize the distribution of the calls during the day and during the week.

Based on those descriptive data, multinomial logistic regression analyses were performed [21–23]. The hour of the incident and the day of the incident were categorized into IH (8:00–16:00) and OOH and weekday and weekend, respectively. Subsequently, three models were made. The dependent variables were the 1–1-2 and 1813 response and the independent variables were IH vs. OOH (model 1), weekday vs. weekend (model 2), and catheter use (model 3). This is because the three independent variables are hypothesized to influence the dependent variable of 1–1-2 and 1813 response. The models were adjusted for confounding and multicollinearity by adding the variables of age and gender. The measures of effect of the independent variables studied on the dependent variable were indicated using the adjusted and unadjusted odds ratio (OR) with a respective confidence interval (CI) of 95% [24–26]. The effect was considered statistically significant for *p* < 0.05 [27, 28]. The data analyses were performed using R version 3.6.1.

## Results

### Patient characteristics, the timing of the incident and response throughout the years

**Fig. 1:**
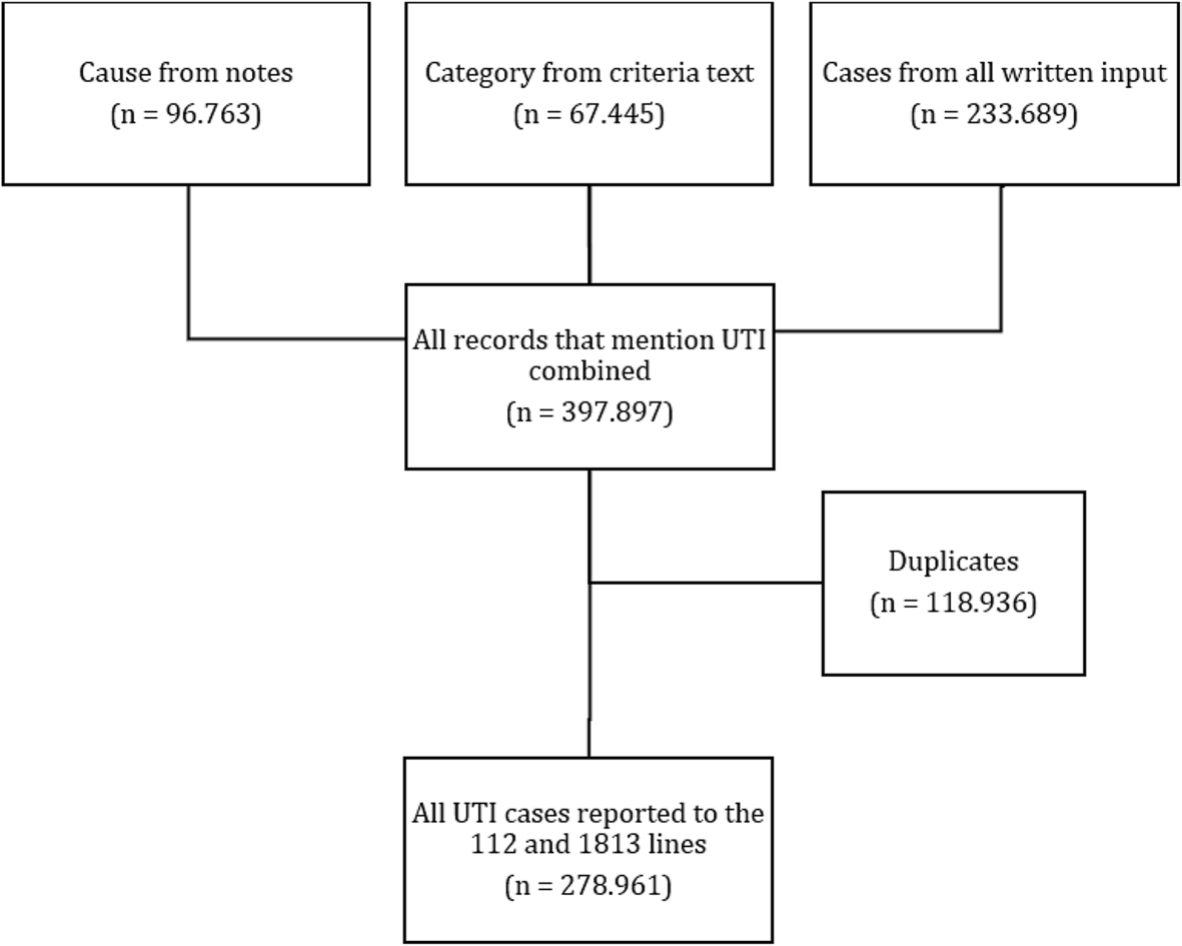
Consort flow chart of data collection flow and retrieval of the final number of records

278.961 telephone calls of people with a UTI or UTI symptoms were reported to the 1–1-2 emergency medical helpline and the 1813-medical helpline between the 10th of December 2013 and the 8th of May 2021. This corresponds to ~ 120 UTI related calls per day and about 3.6% of the total amount of calls to 1–1-2 and 1813 in this timeframe (Fig. 1).

Most callers were women (77.7%) with an average age of 47 years, and 4.9% had a urinary catheter. Furthermore, most people called the 1813-medical helpline (97.5%) and of those patients, the highest proportion ended up being referred to the ED for diagnostics and/or treatment (37.3%). After that, the largest categories are defined as telephone consultation, namely the advice to the patient to visit their GP the next day (20.2%), antibiotic prescription (13.2%), and self-care consultation (12.3%) which – in case of a UTI – generally advises patients to drink sufficient water, to relieve themselves often, and to get some rest [29]. Of the smaller proportion of people calling the 1–1-2 emergency medical helpline, most got a B response (1.5%), which is an urgent, but non-emergency response (Table 2).

**Table 2:** Table of characteristics

	Overall (***N*** = 278.961)
**Age (years)**	
Mean (SD)	47.0 (26.2)
Median [Range]	43.0 [25.0–71.0]
**Gender**	
Female	216,631 (77.7%)
Male	49,781 (17.8%)
**Catheter**	
Yes	13,763 (4.9%)
No	265,198 (95.1%)
**Medical helpline**	
1–1-2	6395 (2.3%)
1813	271,932 (97.5%)
**1–1-2 Response**	
A	1033 (0.4%)
B	4086 (1.5%)
C	334 (0.1%)
D	209 (0.1%)
F	452 (0.2%)
Other	279 (0.1%)
**1813 Response**	
Referral to ED	104,134 (37.3%)
Hospital admission	17,224 (6.2%)
GP next day	56,373 (20.2%)
Selfcare	34,439 (12.3%)
Prescription	36,933 (13.2%)
Other	22,062 (7.9%)

*SD* standard deviation

Out of all patients with UTI symptoms calling 1–1-2, 452 received an F response, which corresponds to 7.1% of all 1–1-2 responses. In contrast, only 6 people calling to the 1813-medical helpline received a 1–1-2 emergency response, which corresponds to only 0.002% of all 1813 responses.

**Fig. 2:**
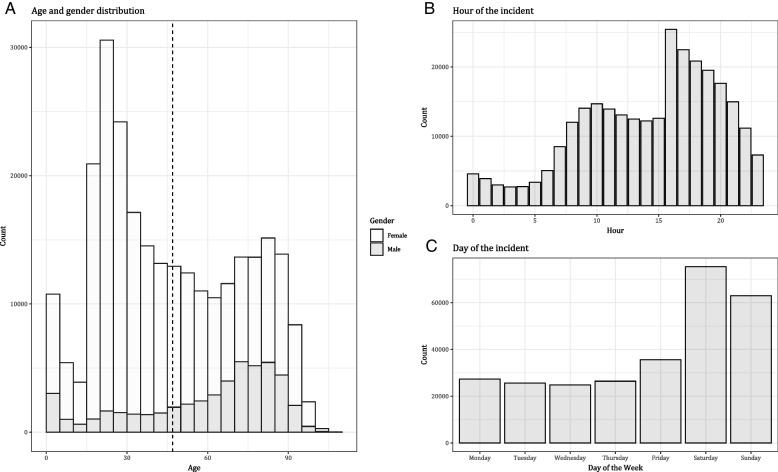
Descriptive figures of patients’ characteristics and timing of the incident, **A** age and gender distribution of the patient group (see in-figure legend), **B** distribution of calls across the hours of the day, **C** distribution of calls during the days of the week

The largest proportion of female patients were between the ages of 15 and 30, but there is also an increase of prevalence in women between 65 and 90. For men, there was only a significant increase in prevalence between the ages of 65 and 90 (Fig. 2A). A high increase in calls between 15:00 and 16:00 was observed, and even a significant proportion of people calling during the IH of GPs (Fig. 2B). Additionally, most people called on Saturdays and a large proportion on Sundays (Fig. 2C).

**Fig. 3:**
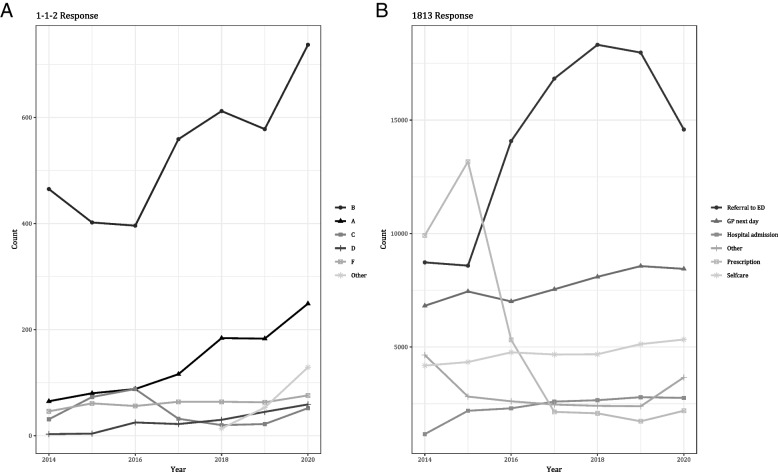
1–1-2 and 1813 response throughout the years 2014–2020, **A** 1–1-2 response categories and **B** 1813 response categories (see in-figure legends)

A similar number of calls each year was observed between the years 2014 and 2020 (Additional file 1: Appendix II). There was a general increase in calls to the 1–1-2 emergency medical helpline, especially in the A and B response categories (Fig. 3A). The number of referrals to the ED increased significantly between 2015 and 2018, just to undergo a decline after 2018. Another category that stood out, was the antibiotic prescription, as this category underwent a drastic decrease between 2015 and 2017. Other categories such as hospital admission and advice to go to the GP the next day or self-care advice, remained relatively stable throughout the years (Fig. 3B).

### Response for the timing of the incident and patient urinary catheter use

The influence of the timing of the incident and urinary catheter use among the patients on the 1813 and 1–1-2 response for UTI was analyzed. Because of the large variance in age and gender distribution (Fig. 2), these two variables were added to the regression models to correct for confounding. Both the adjusted and the unadjusted ORs were calculated, to fully incorporate the effect of age and gender. Three regression models were created, model 1 incorporates the main dependent variable of IH versus OOH calls, model 2 uses the variable of weekday versus weekend and model 3 includes the variable of catheter use. All models incorporated the dependent variables of 1–1-2 and 1813 response (Additional file 1: Appendix I).

**Fig. 4:**
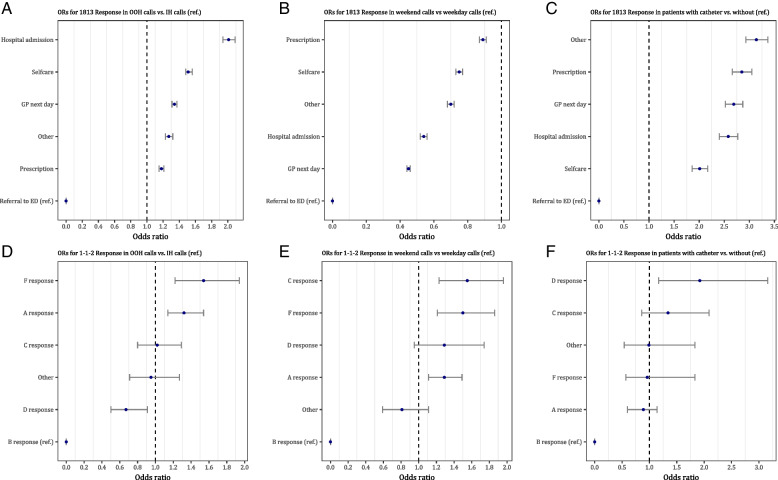
Forest plots of the multinomial logistic regression analysis for 1813 and 1–1-2 response, **A** Odds ratios for 1813 response in OOH calls vs. IH calls (ref.), **B** Odds ratios for 1813 response in weekend calls vs. weekday calls (ref.), **C** Odds ratios for 1813 response in patients with catheter vs. patients without (ref.), **D** Odds ratios for 1–1-2 response in OOH calls vs. IH calls (ref.), **E** Odds ratios for 1–1-2 response in weekend calls vs. weekday calls (ref.), and **F** Odds ratios for 1–1-2 response in patients with catheter vs. patients without (ref.). OOH = out-of-office hours, IH = in-office hours. * *p* < 0.05, ** *p* < 0.01, *** *p* < 0.001

For the 1813 response, a call during the GPs OOH was a highly statistically significant predictor for all responses compared to the referral to the ED response. During OOH, patients are twice as likely to be admitted to a hospital than being referred to the ED compared to the IH calls (OR:2.01; 95% CI:1.94–2.09; *p* < 0.001). Patients were also more likely to be referred to their own GP the next day (OR:1.34; 95% CI:1.31–1.37; *p* < 0.001), to get advice on how to care for the UTI themselves (OR:1.51; 95% CI:1.48–1.56; *p* < 0.001), and to receive antibiotic prescription (OR:1.18; 95% CI:1.15–1.21; *p* < 0.001) (Fig. 4A). For the 1–1-2 response, an OOH call was a statistically significant predictor for an A response (OR:1.32; 95% CI:1.14–1.54; *p* < 0.001) and an F response (OR:1.54; 95% CI:1.22–1.94; *p* < 0.001) compared to a B response. In contrast, patients were less likely to receive a D response (OR:0.67; 95% CI:0.50–0.91; *p* = 0.009) than a B response during OOH calls compared to IH calls (Fig. 4D).

An opposite trend for 1813 response was found during weekend calls compared to calls during the week. During the weekend, patients were less likely to be admitted to the hospital (OR:0.54; 95% CI:0.52–0.56; *p* < 0.001), to be refered to their own GP the next day (OR:0.45; 95% CI:0.44–0.46; *p* < 0.001), to get advice on how to take care of the UTI themselves (OR:0.75; 95% CI:0.73–0.77; *p* < 0.001), and to receive antibiotic prescription (OR:0.89; 95% CI:0.87–0.91; *p* < 0.001) than being refered to the ED (Fig. 4B). For the 1–1-2 response, patients were statistically significant more likely during the weekend to receive an A response (OR:1.29; 95% CI:1.11–1.49; *p* < 0.001), C response (OR:1.55; 95% CI:1.23–1.96; *p* < 0.001) and F response (OR:1.50; 95% CI:1.21–1.86; *p* < 0.001) than a B response compared to a weekday (Fig. 4E).

Having a urinary catheter is a higly statistically significant predictor for all 1813 responses compared to the referral to ED response. For all these responses, patients with cathethers were at least twice as likely to get a response other than referral, namely for hospital admission (OR:2.58; 95% CI:2.40–2.77; *p* < 0.001), referral to one’s own GP the next day (OR:2.69; 95% CI:2.52–2.87; *p* < 0.001), selfcare (OR:2.01; 95% CI:1.86–2.17; *p* < 0.001) and antibiotic prescription (OR:2.85; 95% CI:2.66–3.05; *p* < 0.001) (Fig. 4C). For the 1–1-2 response, the only statistically significant predictor is that patients with a cathether are almost twice as likely to get a D response than a B response than patients without a cathether (OR:1.92; CI:1.17–3.16; *p* = 0.010). Patients with a cathether are also more likely to get a C response (OR:1.50; 95% CI:1.21–1.86; *p* = 0.190) compared to a B response than patients without a cathether, wheras they are less likely to get an A (OR:1.50; 95% CI:1.21–1.86; *p* = 0.495) or an F response (OR:1.50; 95% CI:1.21–1.86; *p* = 0.878) (Fig. 4F).

## Discussion

This retrospective cohort study aimed to describe the characteristics and response plan of a large amount of UTI patients calling the EMS in Copenhagen. Descriptive statistics for age and gender showed a higher proportion of calls from women in their late teens and twenties, as well as in elderly people, which was in line with the evidence on UTI incidence among different age groups that was found in the literature [2]. Additionally, a previous study on OOH contacts in Denmark found that females and age groups above 81 years old and between 0 and 18 years old were more likely to contact the services than males and other age groups [30], which possibly partially explains the similar results that were found in this study. Similarly, the distribution of calls during the day and during the week is possibly due to the fact that most UTIs do not require an emergency response and is therefore in line with the total amount of calls answered by the OOH 1813-medical helpline in the Copenhagen. This shows a higher number of calls during the GPs OOH, which is between 16:00 and 8:00, as well as during the weekend when GPs in Denmark are closed.

Furthermore, a significant proportion of patients calling 1–1-2 received a non-emergency (F) response, whereas only a small proportion of patients calling 1813 received an emergency response (Table 2). This means that dispatchers are successfully able to switch between both systems, leading to the most accurate response for the patient. These findings contribute to the evidence that having two systems in a joint dispatch center is in favor of the patients’ wellbeing and the precision in their received response.

After assessing the dispatch response to UTI calls over the years, the overall number of 1–1-2 calls was found to have increased, particularly the A and B response categories, which means that there is an increasing amount of people with UTI who received an emergency or urgent response. Furthermore, the 1813 response showed some changes over time, particularly in the referral to ED and prescription categories. While the number of patients receiving antibiotic treatment over telephone consultation showed an immense decrease from 2015 onwards, more patients were being referred to the ED as what is assumed to be a response to that trend. This means that instead of immediately being given a receipt for antibiotics by the dispatcher, patients are instead being sent to the ED to be evaluated on the presence of a UTI first, receive a urinary dipstick, and might be treated with specific and targeted medication. This trend is in line with the increased emphasis on antimicrobial stewardship, the emergence of a non-antimicrobial approach to treating uncomplicated UTIs [29], as well as an executive order on antibiotic treatment from the Danish Health Authorities to all GPs in Denmark in 2012 [31], which implicated that GPs were only allowed to prescribe a select number of antibiotics to their patients. This guideline was not fully implemented at the 1813-medical helpline until a year after the start of its operation, which explains the vast decrease of UTI prescription over the telephone between 2015 and 2016. Currently, only patients over 65 years old with recurrent UTI and demonstrable access to results of a previous urinary dipstick with subsequent treatment are receiving antibiotic prescription over telephone consultation, given they are not seriously ill with symptoms such as fever or nausea and there is no blood in their urine [4]. Also, the decrease in referral to the ED response in 2020 is hypothesized to be a result of downscaled care in the ED due to the COVID-19 pandemic. This is in line with previous reports on decreased diagnoses of cancer and cardiac disease in Denmark [32, 33].

Compared to GP IH, patients are less likely to be referred to the ED during OOH than any other response. A possible explanation for this observation is that during IH, patients are supposed to call their GP first, instead of immediately contacting the 1813-medical helpline. During OOH, proportionally more people call the OOH 1813 number. This means that the situation is either more severe, that the patient cannot wait until the next day and needs to be admitted immediately, or the situation is evaluated by the dispatcher as less severe than the patient originally thought, which means the patients are more likely to receive a telephone consultation with the advice of self-care or to go to their GP the next day.

The opposite effect was found for weekend calls compared to a call during the week, which means that patients are more likely to be referred to the ED than to receive any other response. This can possibly be explained by the fact that on Saturday, patients cannot be told to visit their GP the next day and consequently, they are more often sent to the ED for evaluation. Finally, the use of a urinary catheter is considered a comorbidity, which means it requires a different and personalized response. Therefore, patients were more likely to receive the ‘other’ category, which is a compilation of the more uncommon response categories. The second highest response category was that of antibiotic prescription, which is in line with our expectations, as catheter users often experience complicated UTIs and are therefore at a higher chance of having recurrent UTIs [2]. This also explains why patients with catheters are more likely to be admitted to the hospital than being referred to the ED compared to patients without a urinary catheter.

Furthermore, the finding that there are more emergency (A) responses dispatched during OOH calls might be due to coincidence, but it might also be caused by the fact that some people want to wait until the next day to visit their GP first, but get more severely ill in the meantime. On the other hand, the fact that more people receive a non-emergency (F) response, might be related to the 1813-medical helpline. More people might call 1–1-2 instead of 1813 during their GPs OOH, which more often results in a non-emergency response. In contrast, D responses are less likely to be given than B responses in OOH compared to IH, because this type of transport is planned and does not require any medical supervision, which can possibly wait until the next day.

After comparing weekday and weekend calls, surprisingly similar numbers in terms of A and F response were found, which might be explained in the same manner as described in the previous paragraph. However, an increased likeliness to receive a C or D response during the weekend compared to weekday was also found, which might underlie the increased need for planned transport, both monitored and unmonitored during the weekend compared to the week. Patients with a urinary catheter were more likely to receive a planned transport response (C and D) compared to an urgent response (B). This is potentially caused by the patients’ underlying comorbidities for which they need a catheter, which can immobilize them which makes them require (monitored) transport to the hospital for evaluation.

### Implications for the EMS in Copenhagen

To the authors’ knowledge, this is the first study looking at data on the response to people calling with symptoms of UTI of both the emergency medical number and the OOH-medical helpline of a region. The EMS in Copenhagen has its exceptional structure of operating both those number in the same dispatch center, which means that the data was retrieved from one place with all telephone records. This provides many opportunities for the research department at the EMS in Copenhagen to study other types of incidents and diseases which are often handled at the dispatch center.

This study was particularly explorative in its nature and the outcomes gave us more understanding about the patients with UTI calling the EMS and the prehospital response given to these patients. This data can be used to monitor the demand of patients calling with UTI symptoms and whether they got an accurate response based on the dispatch guidelines, indicating a suspected diagnosis for the patient. It is important to evaluate current trends, but also changes over time to assess the need for different resources for patients suffering from UTI. Further research is needed to appraise these numbers compared to the accumulated data on the 1–1-2 and 1813 medical helplines, and to appraise the public health needs of prehospital treatment and care of UTIs.

## Limitations

There are some limitations to the study. Firstly, the collection of data for this study was summarized in a table that is considered big data. This means that there is a lot of information within the dataset, and abnormalities in outcomes are rarely detected. Besides, the data was taken directly from the source, which includes raw patient telephone data, and is therefore not edited before use. This implies that having outliers will be unavoidable. However, in terms of the size of the database, this will not result in any significant changes in regression toward the mean.

Secondly, the data is not based on an official diagnosis, but merely an evaluation from the dispatcher. Therefore, the patients that were included in the database might have a suspected UTI, but there is no way to confirm this diagnosis, as this data is strictly on the telephone call within the dispatch center. This means that some outcomes are inconclusive and that there is a loss to follow-up on the patient and the exact diagnosis and treatment they end up receiving. Finally, this study evaluated caller data on UTIs only, it does not look at the total amount of calls. This means that the outcomes of the descriptive statistics potentially underlie overall trends within the 1–1-2 and 1813 medical helplines.

## Conclusions

This study found that UTI is a common phenomenon at the EMS in Copenhagen. Data from the 1813-medical helpline showed a decrease in antibiotic prescription, as well as a higher referral to the ED and a lower rate of prescription as a response from the 1813- medical helpline. Additionally, on weekends UTI patients were more likely to be referred to the ED than their GP but more likely to referred be their GP the next day during OOH instead of the ED.

## Supplementary Information

**Additional file 1: Appendix I.** Multinomial logistic regression for 1813 and 1–1-2 response. **Appendix II.** Plot for amount of calls each year.

## Data Availability

The data that support the findings of this study are available from Emergency Medical Services, Capital Region of Denmark, Denmark but restrictions apply to the availability of these data, which were used under license for the current study, and so are not publicly available. Data are however available from the authors upon reasonable request and with permission of Emergency Medical Services, Capital Region of Denmark, Denmark.

## Abbreviations

CAD: Computer-Aided Dispatch
CI: Confidence interval
ED: Emergency department
EMS: Emergency Medical Services
GP: General practitioner
IH: In-office hours
OOH: Out-of-office hours
OR: Odds ratio
UTI: Urinary tract infection
