# Supplementary material for: Patient characteristics and dispatch responses of urinary tract infections in a prehospital setting in Copenhagen, Denmark: a retrospective cohort study
Source: BMC Prim Care. 2022 Dec 10;23:319. doi: 10.1186/s12875-022-01915-4 (PMC9736713; doi:10.1186/s12875-022-01915-4)

Appendix

Appendix I – Multinomial logistic regression for 1813 and 1-1-2 response

		Model 1<br>IH vs. OOH call<br>(ref. = IH)				Model 2<br>Weekday vs. weekend call<br>(ref. =weekday)				Model 3<br>Catheter use<br>(ref. = no)			
		**Adjusted OR (95% CI)**	**p value**	**Unadjusted OR (95% CI)**	**p value**	**Adjusted OR (95% CI)**	**p value**	**Unadjusted OR (95% CI)**	**p value**	**Adjusted OR (95% CI)**	**p value**	**Unadjusted OR (95% CI)**	**p value**
**1813 (n=271.165)**	**Referral to ED (ref.)**	.	.	.	.	.	.	.	.	.	.	.	.
	**Hospital admission**	2.01 (1.94-2.09)	<0.001	1.67 (1.61-1.73)	<0.001	0.54 (0.52-0.56)	<0.001	0.64 (0.62-0.66)	<0.001	2.58 (2.40-2.77)	<0.001	8.73 (8.18-9.32)	<0.001
	**GP next day**	1.34 (1.31-1.37)	<0.001	1.21 (1.19-1.24)	<0.001	0.45 (0.44-0.46)	<0.001	0.49 (0.48-0.50)	<0.001	2.69 (2.52-2.87)	<0.001	3.61 (3.40-3.83)	<0.001
	**Selfcare**	1.51 (1.48-1.56)	<0.001	1.40 (1.38-1.44)	<0.001	0.75 (0.73-0.77)	<0.001	0.80 (0.78-0.82)	<0.001	2.01 (1.86-2.17)	<0.001	2.59 (2.42-2.78)	<0.001
	**Prescription**	1.18 (1.15-1.21)	<0.001	1.01 (0.99-1.04)	0.260	0.89 (0.87-0.91)	<0.001	1.03 (1.01-1.06)	0.009	2.85 (2.66-3.05)	<0.001	3.86 (3.62-4.11)	<0.001
	**Other**	1.27 (1.23-1.32)	<0.001	1.04 (1.02-1.08)	0.003	0.70 (0.68-0.72)	<0.001	0.83 (0.81-0.86)	<0.001	3.14 (2.93-3.37)	<0.001	7.17 (6.72-7.64)	<0.001
**1-1-2 (6.393)**	**B response (ref.)**	.	.	.	.	.	.	.	.	.	.	.	.
	**A response**	1.32 (1.14-1.54)	<0.001	1.31 (1.14-1.51)	<0.001	1.29 (1.11-1.49)	<0.001	1.25 (1.08-1.43)	0.002	0.89 (0.60-1.14)	0.495	0.74 (0.54-1.01)	0.055
	**C response**	1.02 (0.80-1.29)	0.874	1.02 (0.81-1.27)	0.897	1.55 (1.23-1.96)	<0.001	1.49 (1.19-1.87)	<0.001	1.34 (0.86-2.09)	0.190	1.30 (0.86-1.96)	0.213
	**D response**	0.67 (0.50-0.91)	0.009	0.64 (0.49-0.85)	0.002	1.29 (0.95-1.74)	0.100	1.27 (0.95-1.68)	0.102	1.92 (1.17-3.16)	0.010	1.65 (1.03-2.63)	0.036
	**F response**	1.54 (1.22-1.94)	<0.001	1.59 (1.29-1.96)	<0.001	1.50 (1.21-1.86)	<0.001	1.37 (1.12-1.67)	0.002	0.96 (0.57-1.61)	0.878	0.68 (0.42-1.09)	0.110
	**Other**	0.95 (0.71-1.27)	0.740	0.98 (0.76-1.25)	0.840	0.81 (0.59-1.11)	0.185	0.87 (0.67-1.13)	0.305	0.99 (0.54-1.83)	0.983	1.33 (0.85-2.07)	0.211

Appendix II – Plot for amount of calls each year

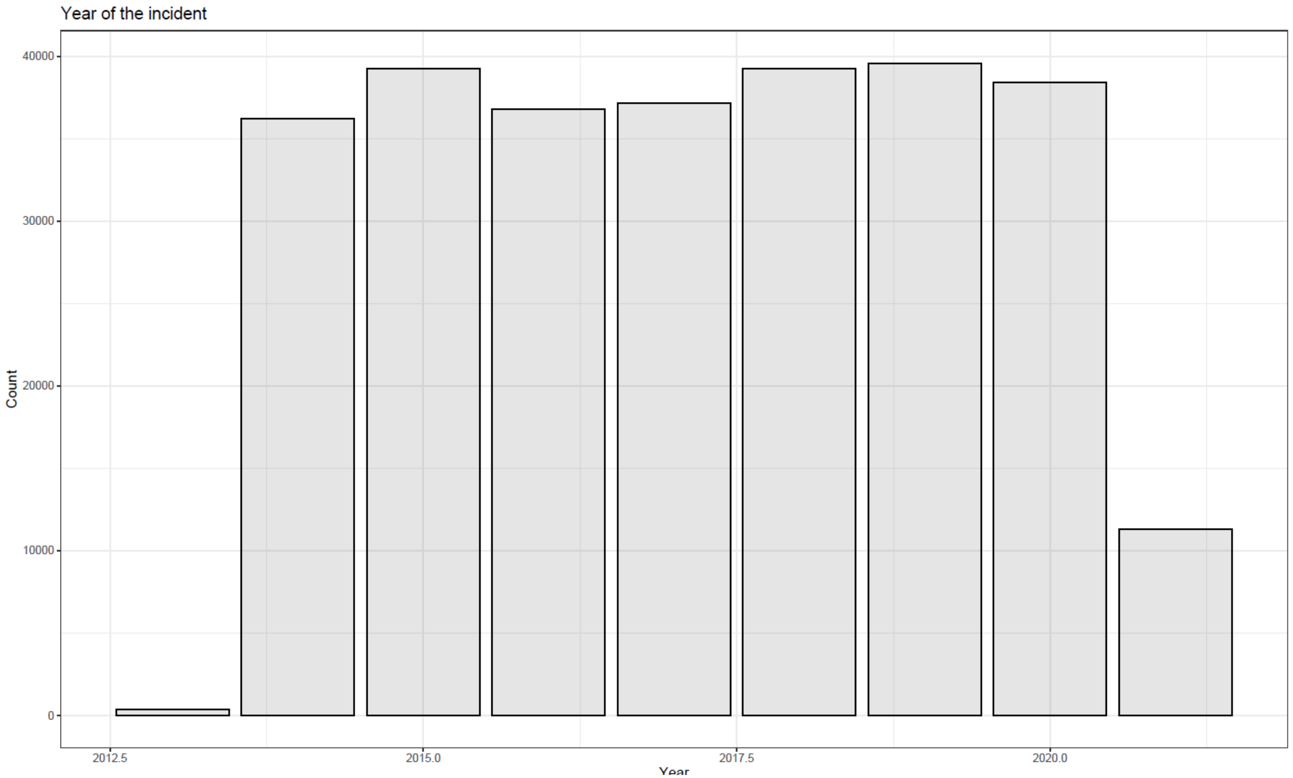

Supplement: Supplementary file 1 — Additional file 1: Appendix I. Multinomial logistic regression for 1813 and 1–1-2 response. Appendix II. Plot for amount of calls each year. [file 12875_2022_1915_MOESM1_ESM.pdf]
